# The Siamese Twins

**Published:** 1836-07

**Authors:** 


### THE SIAMESE TWINS.

[THESE youths, who were brought from Siam in 1829, have been since they left this country to America, and are now in Paris. The following account of them is drawn up by a distinguished young physician of Paris.]

It is a law in fœtal development, that central organs are formed by the union of lateral and similar organs. Thus the sternum is produced by two lateral sternums uniting in the median line, and this explains the remarkable conformation of these twins. Previous to the union of the two halves of the sternum, the right half of the sternum of one fœtus became united to the left half of the other at the lower part, thus connecting the two beings.

This connecting band united them at first face to face, but constant traction has so changed its direction that they are now side by side. Its length above is two inches, below nearly four: from above downwards it measures three inches, and its greatest thickness is one and half inch. It is formed thus: at the lower part of the sternum of each of the twins the xiphoid cartilage turns upwards and forwards, and meets that of the opposite side, between these two cartilages an articulation exists, permitting vertical and lateral motion. The band is thus formed superiorly by the xiphoid appendix, and by some of the cartilages of the ribs, and presents inferiorly the cicatrix of the umbilicus: the cavities of the two chests do not communicate, but the abdominal cavities do, on pushing the finger against the umbilicus into the abdomen when the youths cough, the viscera are felt projecting into the cavity in the band. It is covered by skin, and when the centre is touched both feel it, but on touching either side of the median line only the nearest individual is sensible of it. It has been proposed to divide the band, but if this description be correct,* any incision would open the peritoneum: such a proposal is very disagreeable to the twins, who have often said they have never seen any single individual so happy as they are united.

Their names are Eng and Chang, and they were born in May 1811, in a small village on the coast of Siam, twenty leagues from Bankok, of Chinese parents. They have the Chinese features; the internal angles of the eyes slightly drawn down, skin yellow, and hair very black: they are extraordinarily alike, only Eng is a little larger and stronger, and Chang appears to rest more willingly on his brother. They are five feet in height, well proportioned, and of great muscular strength: they are very agile, they walk and run rapidly, and can swim as well as a single person. Their intellectual faculties are well developed. They understand English and speak it perfectly, but they have forgotten their native tongue, which is not to be wondered at, as they never speak to each other except sometimes asking a question. They have both an equal knowledge of English. Two persons have endeavoured to converse separately with each at the same time, but both turn invariably to one speaker and converse alone with him. They suffered from ague in America: the attack commenced at the same time in both, and the stages of the disease exactly corresponded, so that they experienced rigors, heat, and sweating at the same moment. Chang also had pain in his side, during which, his brother was uncomfortable, and when Chang was being bled, Eng felt indisposed. Their taste for food, for persons, and things, is similar; what pleases one, pleases the other: they both experience hunger and thirst, go to sleep and wake at the same instant: one is never awake whilst the other sleeps, and to wake them it is only necessary to touch one. During sleep they often change their position by one rolling over the other, without waking. There is the utmost uniformity in their motions, as if both were influenced by one will. They have never been known to be angry with each other: the one who wishes to perform any act, makes no sign to the other, who, notwithstanding, concurs without the slightest hesitation,

* M. Coste arrives at a different conclusion, and in a note addressed to the Academy of Sciences states his belief that an operation for their separation offers the greatest chance of success. *Gazette Médicale,* 9 *Jan.* 1836.

and acts as the will of the first determines. This similitude of tastes, conformity of thoughts, and striking harmony of movements, can hardly be explained by mere juxta-position. The great likeness of twins, their brotherly friendship, and other moral and physical analogies are well known; but these Siamese youths from their accidental union are more than twins, and although they form two perfectly distinct beings, they appear most frequently to think, act, and move as one individual.

*Journal Hebdomadaire des progrès des Sciences Médicales,* 2 *Janvier,* 1836.

